# Supplementary material for: Are carbon emissions trading and green financial instruments synergistic? -Comprehensive quantitative research based on content analysis
Source: PLoS One. 2024 Mar 7;19(3):e0298601. doi: 10.1371/journal.pone.0298601 (PMC10919601; doi:10.1371/journal.pone.0298601)
Supplement: S1 Data — (ZIP) [file pone.0298601.s001.zip › Data/地级市Z_F工作报告.zip!/┴╔─■/╙¬┐┌╩╨/└┤╫╘╣1⁄2╓┌║┼í╛┬φ┐╦╩2╛▌═°í┐_2007.docx]

各位代表：

　　现在，我代表市人民政府向大会报告工作，请予审议，并请政协委员和列席会议的同志提出意见。

　　一、2006年工作的回顾

　　2006年，我们在省委、省政府和市委的正确领导下，紧紧依靠全市各族人民，团结奋进，真抓实干，较好地完成了市十三届人大四次会议确定的各项任务，实现了“十一五”的良好开局。全市完成地区生产总值457.7亿元，比上年增长18.9%；全社会固定资产投资290亿元，增长31.2%；财政一般预算收入20.5亿元，增长25.1%；城市人均可支配收入10135元，增长12.5%；农民人均纯收入5001元，增长11.1%。

　　农业和农村经济全面发展。全年完成农业总产值80.1亿元，增长11.3%；粮食总产量72.1万吨，增长6.8%。农业结构调整步伐加快，落实订单农业85万亩，水果、水产和畜牧业产值占农业总产值比重达到75.5%。农业产业化水平不断提高，新建、续建产业化龙头企业项目34个。农业标准化稳步推进，无公害农产品种植面积达到78.2万亩，增长42.2%；无公害、绿色、有机食品认证数量达到75种。科技兴农取得新成效，引进新品种41个，推广新技术10项，建设科技示范基地10个。机械化耕作面积达到165.5万亩，增长10.3%。市、县和区域三级动物疾病防控体系已经形成。农业生产条件不断改善，新增和改善灌溉面积11.8万亩，治理水土流失面积8.6万亩。植树造林7.2万亩，集体林权改革完成68.5%。

　　“工业发展年”活动成效显著。全年开工建设投资1000万元以上工业项目293个，其中5000万元以上90个，亿元以上39个；完成工业固定资产投资200.1亿元，增长39.3%；规模以上工业增加值202.1亿元，增长29.5%；新增规模以上工业企业186户，超额完成“22215”目标任务。鞍本集团营口新厂、五矿中板公司宽厚板、华能电厂二期工程等重大项目建设进展顺利。工业经济运行质量不断提高，实现规模以上工业总产值731.9亿元，增长35.4%；利税56亿元，增长39.8%。冶金、石化、装备制造、镁质材料、纺织服装、新型建材六大产业集群的主导作用初步显现，实现产值594亿元，占规模以上工业总产值的81.2%。科技创新能力不断增强，新建企业研发中心18个，实施500万元以上技改项目139个，重点技术创新项目58个，实现高新技术产业增加值25.6亿元。全市创省以上名牌产品33种，“银珠”牌锦纶66长丝、“诺的斯卡”牌钢琴荣获2006年中国名牌产品称号，“千里明”系列产品荣获中国驰名商标。推广资源节约和综合利用项目36个，万元地区生产总值能耗下降4.5%，主要污染物排放总量控制在省计划指标以内。企业改革力度不断加大，实施市考核企业改制和盘活资产项目69个，基本完成省下达的国企改革任务。民营经济快速发展，实现企业增加值320亿元，增长28.2%，上缴税金26亿元，增长23.8%。

　　外向型经济发展水平进一步提高。全年新批外商投资企业150户，外商直接投资1.01亿美元，增长28.6%。落实内联项目262个，资金实际到位额123亿元。对外贸易稳定增长，全年完成进出口总额16.8亿美元，增长11.3%，其中出口11.72亿美元，增长16.1%。劳务输出5215人，增长37%。新增境外投资企业4家。港口功能不断完善，鲅鱼圈港区四期工程、A港池、仙人岛港区开发顺利推进，全年完成港口吞吐量9477万吨，增长25.7%；集装箱运量101万标箱，增长28%。

　　沿海经济带建设步伐加快。辽宁（营口）沿海产业基地总体规划及各专项规划均已形成，基础设施建设和项目开发取得可喜成果。完成道路路基工程110公里，给排水工程180公里，电力线路35公里，回填土地14平方公里；中冶京诚公司中试基地等12个项目已开工建设，基础设施建设及入区项目完成投资15亿元。开发区先导作用进一步增强，实现地区生产总值135亿元，增长30%；固定资产投资141.2亿元，增长37%；开工建设1000万元以上项目46个；实际利用外资4500万美元，增长28%；出口创汇5.2亿美元，增长15.6%。高新区新开工500万元以上项目38个，实现技工贸总收入44.8亿元，增长1倍。船舶工业园万吨级货轮顺利下水，实现了营口造船业历史性突破。镁质材料产业基地、仙人岛能源化工区、冶金和重装备制造工业区建设也都取得了实质性进展。

　　第三产业保持较快增长。全年实现社会消费品零售总额122.6亿元，增长14.6%。城市商业网点和“万村千乡工程”建设成效显著，新增社区商业网点580个、农家店375家。全年实现旅游总收入17.47亿元，增长20.1%，顺利通过了中国优秀旅游城市验收。邮电通信业务总量25亿元，增长26.8%。保险费总收入10.6亿元，增长14.5%。金融环境进一步改善，金融机构存款余额453.8亿元，增长12.9%；贷款余额325.3亿元，增长24.2%；不良贷款比率比年初降低2.9个百分点；中小企业信用体系累计为企业提供担保贷款11.4亿元。

　　城乡基础设施和环境明显改善。年初确定为市民办的15件实事基本实现年度计划，完成棚户区改造30.1万平方米，接转上年40万平方米，安置动迁居民8000余户。重点工程全面推进，污水处理一期、中水回用和西部热电厂相继投入使用。完成交通建设投资33.2亿元，新增公路92.9公里。公开出让土地23.24公顷，成交额1.49亿元；出让采矿权26宗，获得采矿权价款1278万元。积极开展国家卫生城和环保模范城创建活动，加强城乡环境卫生整治，城市管理水平不断提高，人居环境进一步改善，大气环境质量保持在二级标准之内。

　　就业和社会保障体系建设进一步加强。全市新增实名制就业8.8万人，城镇登记失业率控制在4.8%。农村剩余劳动力转移就业4.8万人，实名制培训农村剩余劳动力5.2万人，培训下岗失业人员3.8万人。企业养老保险新增扩面3.3万人，总数达到36.6万人。医疗保险新增参保人数9.5万人，总数达到42.6万人。城市低保人数和农村低保人数分别达到9.1万人和3万人，全年发放城乡居民最低生活保障金10788万元。

　　社会各项事业全面进步。教育事业取得新成果，四个区在全省首批实现“双高普九”，农村初中全部进入省标准化初中行列。建立贫困家庭学生就学资助体系，严格落实省定“两免一补”政策，促进了城乡义务教育的发展。大力实施文化精品工程，城乡群众文化生活日益活跃。西炮台晋升为国家级文物保护单位。广播电视网络和数字化进程加快，完成了69个行政村广播电视“村村通”任务。城镇医疗卫生体制改革稳步推进，疾病预防控制和卫生监督工作得到加强。新型农村合作医疗全面推开，农民参合率80.2%，农民看病难、看病贵问题得到缓解。人口和计划生育工作不断取得新成绩，人口自然增长率控制在1.15‰以内。广泛开展群众性体育活动，竞技体育水平有所提高， 在省十运会上我市金牌总数名列第四。以推进“双十工程”为重点，“双拥共建”取得新成果，国防教育、民兵预备役和老龄等项工作得到进一步加强。

　　民主法制和政府自身建设不断加强。认真执行市人大及其常委会的决议，及时向市政协通报政务情况，自觉接受市人大的法律监督、工作监督和市政协的民主监督，广泛听取各民主党派、工商联、无党派人士和人民团体的意见和建议，全面落实党的民族、宗教和侨务政策。全年共办理人民代表建议和政协委员提案452件，办复率100%，满意率99.6%，办结率72.3%。积极开展“五五”普法宣传，严厉打击各种犯罪活动，社会治安综合治理和治安防控体系建设成效显著，刑事案件发案率下降8.5%，切实增强了人民群众的安全感。妥善处理群众来信来访，信访处结率80%，市民投诉办结率90%以上。加强安全生产工作，安全生产事故下降6.8%，死亡人数下降11.4%。继续削减和调整行政许可项目，进一步规范政府部门职能，行政效能不断提高，我市进入全国投资环境百佳城市行列。

　　过去的一年里，全市国民经济和社会各项事业发展取得了显著成绩，这是全市人民发奋图强、共同努力的结果，是市委正确领导、市人大依法监督、市政协民主监督的结果，是各民主党派、工商联、无党派人士、人民团体、驻营部队和中省直单位帮助、支持的结果。在此，我代表市人民政府，向全市广大干部群众，向所有关心、支持营口建设的同志们、朋友们表示崇高的敬意和衷心的感谢！

　　在肯定成绩的同时，我们也应清醒地看到，全市经济和社会发展仍然存在一些不容忽视的问题：一是农副产品加工率较低，农村工业化进程缓慢。二是工业经济增长方式还比较粗放，部分企业自主创新能力不强。三是利用外资规模不大，项目质量不高。四是财政收入占地区生产总值比重较低，供需矛盾突出。五是劳动力结构性矛盾突出，就业压力增大。六是少数公务员作风不实，服务意识不强。对此，我们要在今后的工作中，采取有效措施，认真加以解决。

　　二、2007年的主要工作任务

　　2007年是本届政府任期的最后一年，做好今年工作，全面完成本届政府的各项目标任务，对实现营口老工业基地的全面振兴至关重要。我们必须以良好的精神状态、创新的发展思路、科学的执政方式、务实的工作作风，努力开创营口改革开放和现代化建设的新局面。

　　按照市委十届三次全会的部署和要求，今年工作总的指导思想是：以邓小平理论和“三个代表”重要思想为指导，全面贯彻落实科学发展观，继续实施以港兴市、工业强市战略，着力抓好加快新型工业化进程、推进社会主义新农村建设和构建和谐营口三大重点任务，不断深化改革、扩大开放，推进科技创新，优化经济结构，做大经济总量，提高增长质量。同时，大力加强社会各项事业，切实关注和解决好民生问题，推动经济社会又好又快发展，为实现营口老工业基地全面振兴奠定坚实基础。

　　2007年全市经济社会发展的主要目标是：地区生产总值540亿元，比上年增长18%；全社会固定资产投资362亿元，增长25%；实际利用外资1.3亿美元，增长30%；外贸出口13.5亿美元，增长15%；社会消费品零售总额138亿元，增长13%；地方财政一般预算收入25.6亿元，增长25%；城市人均可支配收入和农民人均纯收入均增长10%以上；城镇登记失业率控制在4.5%以下；人口自然增长率控制在1.81‰以内；社会各项事业与经济建设协调发展，城市文明程度不断提高。

　　为实现上述目标，要重点抓好以下10个方面工作：

　　（一）突出港口牵动作用，加速沿海经济带建设

　　进一步做大做强营口港。继续增加资金投入，确保四期、A港池等重点工程进度。加快仙人岛港区建设，保证按计划投入使用。搞好对沙鲅铁路的跟踪服务，确保年内开工。不断完善覆盖东北腹地的陆港口岸系统，建立货源建设奖励基金，促进货源基地开发。积极开辟对外航线，扩大外贸出口运量。进一步加强港口运输服务，建设口岸联检大厅，实行“五加一”工作制，完善“大通关”口岸体系。力争10月份实现亿吨大港目标，全年完成吞吐量1.14亿吨，增长20%；集装箱运量122万标箱，增长20%。

　　加快推进开发区建设。要创新发展模式，坚决防止低水平、高污染建设和粗放式增长，进一步提高项目质量。加大基础设施建设投入，不断完善城市功能，努力打造适合人居、适合投资、具有较强竞争力的先导区和新兴产业集聚区。全年地区生产总值和实际利用外资等主要指标增幅确保30%以上。

　　强力推进沿海产业基地建设。要坚持基础设施建设与项目开发并重的原则，确保6月底完成一期40平方公里综合管网铺设，9月底完成主干道黑色路面及部分绿化工程，加快5万吨污水处理厂、华能营口热电厂等重点项目建设。加大招商引资力度，积极推进富士康电子制造、中国五矿园区开发等重点项目的实施，全年新开工投资5000万元以上内联项目和500万美元以上外资项目40个，完成固定资产投资50亿元，确保15个项目投产。

　　加快推进重点园区建设。高新区要增强技术创新和科技孵化能力，加强高新技术项目的开发和引进，全年开工1000万元以上项目28个，主要经济指标增幅50%以上。仙人岛能源化工区要坚持总体规划与专项规划同步推进，开发建设与招商引资同步推进，近期重点抓好土地、资金等要素的安排，确保基础设施上半年开工建设。大石桥南楼等省级开发区要在基础设施建设和项目入驻上实现新突破，主要经济指标增幅高于全市平均水平。

　　（二）深入开展“工业发展年”活动，加快工业强市进程

　　着力推进项目建设。全面实行项目责任制，促进在谈项目早签约，已批项目早动工，在建项目早投产。要积极推进鞍本集团营口新厂、中冶京诚公司中试基地、五矿中板公司宽厚板等投入规模大、产业层次高、带动能力强的重大项目建设，大力培育和扶持中小企业发展。年内完成规模以上工业增加值263亿元，工业固定资产投资260亿元，新开工千万元以上项目200个，新增规模企业150户。

　　大力促进产业集群发展。围绕打造镁质材料、精品钢材和钢铁装备制造业基地、加快原材料工业产业升级、提升轻纺工业竞争力和促进区域经济协调发展，集中生产要素和各类资源，着力培植六大产业集群，做到总量做大，产品做优。年末除新型建材以外的五大产业总产值全部超过100亿元。

　　进一步深化企业改革。妥善做好国有企业改革的后续工作，全年实施改革和盘活资产项目40个。强化国有资产监管，确保国有资产保值增值。引导企业从自身实际出发，加快建立现代企业制度。要进一步营造氛围，积极推进民营企业机制创新、管理创新和技术创新，鼓励、支持和引导民营企业快速发展。

　　提高企业创新能力。引导企业加强研发机构建设，推进自主创新，大力扶植具有自主知识产权项目的开发建设，全年实施重点技术创新项目60个、技术改造项目120个，新建市级以上企业研发中心15个，高新技术产业增加值增长30%以上。继续实施名牌战略，力争在国家和省名牌产品录上有更多的营口品牌。

　　大力发展循环经济。淘汰落后生产工艺和设备，推广节能降耗、清洁生产等先进技术，实施一批余热余压利用、热电联产项目，发展节能产品。强化污染企业治理，严把项目审批、市场准入关，限制高耗能、高污染项目建设。加强矿产、土地等资源保护，妥善处理生产、生活垃圾和危险废弃物。全年实施资源节约与综合利用及环保项目40个，万元地区生产总值能耗下降4.5%；主要污染物排放总量减少2.5%。

　　（三）以增加农民收入为重点，全面推进社会主义新农村建设

　　大力发展县域经济。坚持以工业为主导，突出抓好县级工业园区建设，选择有实力、有基础的园区，给予省级开发区的政策支持。积极推进城乡一体化进程，着力抓好熊岳、高坎等15个中心镇建设，完善小城镇功能。切实做好扩大县（市）经济管理权限试点工作，不断探索适合县域经济发展的行政管理机制。加强基层政权建设，认真做好村民委员会换届工作。加大财政投入，财政支农资金用于县域经济发展的比重要高于上年，促进经济发展较快地区更加发展，特别要加大对欠发达地区专项资金的支持力度，到年末县域经济增长速度要高于全市平均水平。

　　以工业理念推动现代农业快速发展。重点抓好优质农产品生产和加工基地建设，大力发展优质水稻、水果、水产、设施蔬菜、畜牧养殖等特色产业。全年粮食作物播种面积稳定在146万亩，订单农业面积确保在90万亩以上，设施蔬菜和水果面积达到22万亩，工厂化养殖面积达到25万平方米，畜牧业占农业总产值比重提高1-2个百分点。集中力量扶持产业化龙头企业，全年新建、续建、扩建投资500万元以上项目20个。继续完善农产品质量检验检测体系，无公害农产品生产面积增长15%。加强农村基础设施建设，新改扩建水源工程175处，新增灌溉面积2万亩。认真落实林业政策，全年完成植树造林4万亩，封山育林5万亩。进一步加大科技兴农力度，提高科技对农业增长的贡献份额。全年引进推广农业科技新品种40个、农村适用新技术10项，建设农业科技示范基地10个。

　　认真实施新农村建设的重点工程。充分发挥政府的主导作用、农民的主体作用和市场机制的基础作用，用好用活国家政策，引导全社会力量参与新农村建设。以“六项整治”和“八项建设”为重点，对村容村貌进行综合整治，进一步改善农村人居环境。组建农民工务工服务机构，加强农民职业技能培训，鼓励和引导农村剩余劳动力向二、三产业转移，全年培训农村剩余劳动力2万人，转移2.5万人。认真落实各级领导包扶责任制，采取贷款贴息等方式，促进东部山区扶贫开发。优化农机装备结构，加快农业机械化进程。密切关注农村饮用水安全，全年解决3.5万人安全饮水问题。大力实施沼气工程，加快农村新能源建设。积极推进农村路网改造，确保完成村村通油路170公里。

　　继续深化农村改革。稳定土地家庭承包经营制度，引导土地经营权有序流转，全面完成集体林权体制改革。扎实推进农村综合改革，强化公共服务职能。扶持发展多种形式的专业合作组织，加强农业信息服务网络建设，为农民提供有效服务。认真落实“三补贴”政策，确保补贴资金及时、足额到位。依法化解村级债务，坚决制止新债发生。全面清理涉农收费，切实减轻农民负担。

　　（四）以招商引资为重点，全面提高对外开放水平

　　进一步加大外引内联力度。改进和完善招商方式，重点推进园区和项目招商，组织好对重点国家和地区的定向招商。坚持外引内联并重，抓住发达地区产业转移和重点企业资本扩张的机遇，促进对内经济技术合作，全年确保引进内联资金160亿元。

　　切实加强招商基础工作。坚持以沿海经济带开发、六大产业集群培植为重点，以企业为主体，把三次产业和社会事业、城乡基础设施建设都纳入对外招商范围，千方百计挖掘项目源，建立多层次的项目库。全年包装重点对外招商项目100个，内联项目200个。要建立完善推进机制，落实工作责任制，强化跟踪服务，促进项目落实。

　　积极扩大对外贸易。坚持以质取胜和科技兴贸战略，扩大机电等高附加值产品出口，优化出口产品结构。加强出口基地和出口专厂建设，建立和完善镁制品、纺织服装、农副产品等8个出口基地，全市出口企业要达到550家，出口额1000万美元以上的企业达到30家。努力开拓国际市场，鼓励和引导有条件的企业境外投资。充分发挥行业协会、商会的自律、协调和培训作用，规范企业行为，有效规避贸易风险。

　　（五）加快发展新兴服务业，推动第三产业升级

　　改造提升传统服务业。进一步优化商业网点布局，重点发展社区零售业，全年新增网点750个。继续实施“万村千乡工程”，新建农家连锁店240家。积极发展信息咨询、中介服务等现代服务业。加强食品、药品和农资等主要商品的市场监管，严厉打击制假售假、欺行霸市等违法行为，努力维护市场秩序。

　　加快发展现代物流业。积极推进营口港保税物流中心、沿海产业物流基地等物流项目建设，加快构筑以港口为龙头，以数字化口岸和电子商务为支撑的现代物流格局，为经济发展提供有力支持和服务。

　　大力发展旅游业。巩固中国优秀旅游城市创建成果，进一步完善各项旅游基础设施，重点开发熊岳、双台温泉和白沙湾浴场、青龙山景区及滨河滨海等旅游资源，全力打造望儿山、西炮台等旅游品牌。积极参与东北地区旅游市场一体化建设，促进旅游接续产业加快发展，全年旅游总收入实现20亿元。

　　（六）加强基础设施建设和城市管理，提升城市功能和品位

　　强化城市规划和管理。坚持科学性、先进性、严肃性相统一，搞好区域性规划的有效衔接，促进主城区与盖州、大石桥两个副中心城市及鲅鱼圈新区规划的协调统一。本着控制总量、盘活存量、提高质量的原则，促进房地产有序开发，加大建筑市场、物业市场和矿业市场的规范整顿力度。加强社区建设与管理，完善社区服务功能。坚持建管并重的原则，强化电力、通讯、市政等公用设施维修、养护和监管，推进城市交通、市容市貌等综合治理，认真解决小街小巷、非物业管理小区和城乡接合部环境卫生脏乱差问题，搞好城市供暖和城市绿化，努力改善人居环境。

　　集中力量为市民办好实事。以解决关系群众切身利益问题和促进社会事业发展为重点，努力办好“十件实事”，启动“五大工程”。1、建设廉租住房2万平方米；2、新建、改造市区主要道路7条；3、改造旧住宅小区7个；4、建设老干部健身中心；5、改扩建营口市养老院；6、建设营口市社区服务中心；7、建设农民工培训示范基地；8、建设村级组织活动场所和农村文化信息资源共享系统；9、全面开展农村乡镇和城市社区医生培训；10、建设县、乡镇客运站8座。启动辽河大街西段改造、海滨带状公园、石门水库供水、物业管理系统化建设和新农村建设规划“五大工程”。同时抓好供电、供热等重点项目实施，建设好滨海大道、营柳路等道路工程，继续实施5万平方米以下连片棚户区改造。

　　稳步推进公用事业改革。认真贯彻落实省政府《关于加快全省公用事业改革的意见》，以城市集中供热、供水、供气特许经营权转让为重点，积极稳妥推进市政公用系统市场化步伐。

　　（七）深化财税金融体制改革，确保财税金融健康平稳运行

　　进一步抓好财源建设。加强对重点税源企业的扶持和监控力度，积极推进金税、金财工程建设。坚持依法治税，切实抓好重点企业、重点产品和重点税种的税收征管，做到应收尽收。加强非税征管，整合财力资源，增加政府可用财力。

　　优化财政支出结构。深化部门预算编制、国库集中支付、政府采购、非税收入收缴等项改革，完善国库单一账户和国库动态监控体系，依法规范政府采购行为，强化国有资产监管。坚持确保重点的原则，严格控制消费性支出，大力压缩会议费、出国考察费、车辆购置费等一般性支出，不断提高财政资金使用效益。

　　加强金融生态环境建设。引导和支持金融机构深化改革，推进城市商业银行增资扩股和完善法人治理结构，做好城乡信用社改革验收及后续工作。进一步完善中小企业信用体系，扩大担保规模。拓宽直接融资渠道，积极争取国家政策性银行和外埠股份制银行的信贷支持，推进企业上市融资，大力发展保险事业。

　　（八）高度关注民情民生，努力构建和谐营口

　　千方百计扩大就业和再就业。加强对就业和再就业的政策扶持，建立和完善政府主导、市场引导的社会化就业服务体系。大力推进普惠制培训，切实做好零就业家庭子女免费培训和农村剩余劳动力技能培训，加强“ 4050”人员和城镇失业人员的培训，全年培训城镇下岗失业人员1.4万人。多渠道开发就业岗位，年内新增就业6.5万人。加快劳动关系协调机制建设，认真解决拖欠农民工工资问题，切实保障农民工的合法权益。

　　不断健全社会保障体系。继续完善城镇职工养老、医疗等基本社会保险制度，不断提高基金收缴率和企业参保率。完善最低生活保障、医疗救助和被征地农民基本生活保障制度，做到低保对象应保尽保，各类困难群众及时得到救助。

　　全面推进法治营口、平安营口建设。深入开展“五五”普法宣传教育，实施民主法治示范村、法治社区等创建活动。继续加强社会治安综合治理，严厉打击各种刑事和经济犯罪，完善覆盖城乡的社会治安防控体系，增强驾驭治安局势和维护社会稳定的能力。全面推进“三基”工程建设，不断提高公安队伍的整体素质。加强安全生产监管，严格落实安全生产责任制，坚决遏制重特大事故发生。认真贯彻《信访条例》，进一步落实信访工作责任制，引进调解机制，化解行政争议，努力把各类矛盾纠纷解决在萌芽状态。

　　（九）大力发展社会各项事业，不断提高社会文明程度

　　坚持教育优先发展。认真贯彻落实《义务教育法》，确保中小学生均公用经费达到省定标准。积极兴办农村九年一贯制寄宿学校，完善贫困家庭学生就学资助体系，农村初中辍学率控制在2.75%以下。全面实施素质教育，高水平、高质量地普及九年义务教育，力争在全省率先整体实现“双高普九”工作目标。大力发展职业教育、高等教育，规范发展民办教育，办好特殊教育和幼儿教育，学前三年普及率达到85%以上。

　　努力构建健康和谐的文化环境。继续办好望儿山母亲节等重大节日和纪念日的公益性文艺演出活动，活跃城乡文化生活。完成60个行政村有线电视村村通工程，着手在全市41个乡镇、646个行政村建立文化服务站。深入开展“扫黄打非”工作，繁荣和净化文化市场。努力发展文化产业，构建以公有制为主体、多种所有制共同发展的文化产业格局。

　　积极推动卫生、体育等项事业健康发展。建立健全公共卫生服务体系、医疗救治体系和急诊急救网络，在市区建立四个社区卫生服务中心。整合卫生资源，加快市中心医院建设，确保年内投入使用。启动新型农村合作医疗网络系统，简化报销程序，完善服务功能。全面加强人口和计划生育工作，统筹解决人口问题，切实稳定低生育水平，努力提高出生人口素质，全年人口出生率控制在6.07‰以下。坚持群众体育、竞技体育协调发展，不断提高全民身体素质。

　　加强人才队伍建设。采取委培、联合办学和开展技能竞赛等方式，努力提高各级各类人才的整体素质。落实人才激励政策，加强和完善人才信息库建设，促进人才合理流动，努力营造人尽其才、才尽其用的良好社会环境。

　　加强精神文明建设。积极推进社会主义核心价值体系建设，以“共铸和谐”为主题，深入开展文明城市创建活动，增强公民社会责任感，促进全社会道德水准和文明程度不断提高。继续开展学习日活动，推进学习型城市建设。加强国防教育、国防动员和海防建设，提高应急行动执行力建设，努力推进“双拥共建”工作，力争蝉联全国双拥模范城。

　　（十）加强政府自身建设，不断提高行政执行能力

　　加强亲民型政府建设。坚持为人民服务，对人民负责，让人民满意的行政准则，集中精力抓发展，实心实意解民忧，让群众得到更多实惠。

　　加强法制型政府建设。大力推进社会主义民主政治进程，强化政府组成人员及其公务员的依法行政意识和法制观念。自觉接受人大及其常委会的依法监督和政协的民主监督，认真办理人民代表议案、建议和政协委员提案。倾听各民主党派、工商联、无党派人士和人民团体的意见，落实党的民族、宗教、侨务政策，充分调动各族各界的积极性、创造性。完善民主决策机制，健全重大事项社会公示制度、听证制度和专家咨询制度，保证各项决策更加符合实际，更加符合人民群众的根本利益。

　　加强责任型政府建设。以提高执行力为重点，建立和完善一级对一级负责，层层抓落实，事事求实效的行政管理体制，努力提高行政效能。认真落实行政问责制、责任追究制，对政府部门负责人的决策失误、工作失职等方面问题进行责任追究。

　　加强服务型政府建设。紧紧围绕改善和优化发展环境，坚持转变职能与创新管理方式相结合，做到该管的一定管好，该放的彻底放开。深化行政审批制度改革，推进职能部门审批机制创新。加强和改进目标管理体系，实行科学的绩效考核和评价机制。切实转变工作作风，坚决反对形式主义和虚报浮夸，全面清理整顿各种评比达标活动，下决心精简会议和文件，以更多的时间和精力抓好各项工作的落实。

　　加强廉洁型政府建设。深入开展廉政建设和反腐败斗争，坚决纠正行业不正之风，切实解决好损害群众利益的突出问题，全面推行政务公开，努力从源头上预防和治理腐败。大力发扬艰苦奋斗的光荣传统，积极倡导廉洁从政之风、勤俭节约之风，建设勤政廉政的公务员队伍。

　　各位代表，面对新的形势和任务，市政府将认真履行党和人民赋予的神圣职责，在市委的领导下，紧紧依靠全市230万人民的智慧和力量，奋发进取，不辱使命，确保圆满完成今年的各项目标和任务，以优异的成绩迎接党的十七大胜利召开！
